# Orexinergic Modulation of Spinal Motor Activity in the Neonatal Mouse Spinal Cord

**DOI:** 10.1523/ENEURO.0226-18.2018

**Published:** 2018-11-08

**Authors:** Sukanya Biswabharati, Céline Jean-Xavier, Shane E. A. Eaton, Adam P. Lognon, Rhiannon Brett, Louisa Hardjasa, Patrick J. Whelan

**Affiliations:** 1 Hotchkiss Brain Institute, University of Calgary, Calgary, Alberta T2N 1N4, Canada; 2 Department of Comparative Biology and Experimental Medicine, Faculty of Veterinary Medicine, University of Calgary, Calgary, Alberta T2N 1N4, Canada

**Keywords:** central pattern generators, movement, spinal cord

## Abstract

The role of orexin during development, and especially in terms of spinal cord function, is not well understood. It is for this reason that we focused on the network actions of orexin during the first week of development. We found that orexinergic fibers were present in the lumbar spinal cord of postnatal day 0 (P0) to P3 mice. The fibers were expressed mainly in the dorsal horn, but occasional fibers were observed in the ventral horn. Both orexin (OX) A and OXB increased the motoneurons (MNs) tonic neurogram discharge. However, only OXA was found to significantly increase spontaneous bursting activity and the frequency of fictive locomotor bursts. We show that OXA is able to act directly on MNs. To test the contribution of the recurrent MN collaterals, we blocked the nicotinic cholinergic drive and observed that OXA retained its ability to increase fictive locomotor activity. Additionally, we recorded neurograms from ventral lateral funiculi, where OXA had no effect on population discharge. These effects were also confirmed by recording from descending commissural interneurons via patch recordings. The loci of the effects of OXA were further investigated in a dorsal horn-removed preparation where OXA also shows an increase in the discharge from ventral root neurograms but no increase in the frequency of spontaneous or fictive locomotion burst activity. In summary, multiple lines of evidence from our work demonstrate the robust effects of orexins on spinal cord networks and MNs at the time of birth.

## Significance Statement

Orexinergic cell recordings show that the cell population is highly active during locomotion. In this study, we show that during the first neonatal week, orexin fibers and receptors are present within the spinal cord and that orexins can affect spinal locomotor circuitry. We examine their effects on motoneuron firing and commissural interneurons. Overall, our work establishes that the orexin family contributes to the control of spinal motor function.

## Introduction

Orexin (OX) neuropeptides were discovered in 1998 and consist of OXA and OXB (de Lecea et al., 1998; Sakurai et al., 1998). OXergic neurons are confined to the lateral hypothalamus (LH), an area associated with a variety of goal-directed actions, including locomotor initiation (Li et al., 2014; Sakurai, 2014). OX modulates excitatory and inhibitory actions on neurons through G-protein-coupled actions (G_αq_, G_αi/o_ G_αs_) via OX1 and OX2 receptors (Sakurai et al., 1998; Kukkonen, 2013; Lebold et al., 2013). In the motor control field, OXs are hypothesized to be important for goal-directed motor behaviors such as reward acquisition (e.g., food) and escape responses (Sakurai et al., 1998; Kim et al., 2017). Indeed, recordings from OXergic areas in the LH show that spike activity is highest during exploratory locomotion such as foraging and eating (Mileykovskiy et al., 2005), and locomotor activity is also increased following intracerebroventricular administration of OX (Hagan et al., 1999). Within the spinal cord, orexins have been shown to have an inhibitory effect on afferent transmission (Grudt et al., 2002; Shono and Yamamoto, 2008; Jeon et al., 2015), which is consistent with its reported antinociceptive function (Bingham et al., 2001). Work on the cat has shown that LH stimulation produces an initial EPSP followed by a late IPSP in lumbar motoneurons (MNs) through OX release in the spinal cord (Yamuy et al., 2004), which is supported by anatomic evidence of OX fibers within the spinal cord (van den Pol, 1999). Indeed, OXs have been proposed to be primarily associated with somatosensory function, based on evidence showing high c-Fos staining only during motor activity (Torterolo et al., 2003). These studies cited thus far have all been performed in adult preparations.

In contrast to the adult, studies of the actions of OX on motor systems during development is scarce; this is important to understand since neuromodulatory classes cannot only acutely activate spinal networks but also contribute to establishing network connectivity (McLean et al., 2000; Whelan, 2003). The application of OX to LH neurons produces a profound increase in spontaneous postsynaptic potentials at birth in slice preparations and in cultured embryonic hypothalamic cells (van den Pol et al., 2001). As early as embryonic day 20 (E20) in the rat, OX-immunoreactive (OX-IR^+^) axons are observed throughout the brain, including the spinal cord, and within the brainstem hypoglossal MNs are depolarized by bath application of OXA (van den Pol et al., 2001). OXA and OXB cell bodies are located in the LH at birth, and indeed OXs are present by at least E18 to E20 and possibly earlier (van den Pol et al., 2001).

At birth, spinal networks produce spontaneous bouts of activity, which is thought to refine synaptic connectivity within the network (O’Donovan, 1999). The network, and the spontaneous activity generated, is developmentally regulated by descending monoaminergic projections (Branchereau et al., 2002). Thus, OX, given its maturation perinatally, could contribute to acute activation of locomotor networks as a consequence of motivated feeding behavior.

In this manuscript, we test the hypothesis that the activation of orexinergic receptors are capable of acting directly on spinal MNs and networks perinatally. We support this hypothesis by (1) establishing that OXA can directly depolarize MNs and alter intrinsic properties, leading to an increase in excitability; (2) showing that OXA and OXB can increase tonic activity and OXA being capable of inducing slow rhythmic bursts; (3) illustrating that OXA can modulate locomotor rhythms both within the isolated spinal cord preparation as well as the leg-attached preparation, which allowed us to establish effects on muscle activation; and (4) providing evidence that these effects on locomotor activity do not appear to be due to depolarization of commissural descending interneurons within the ventral spinal cord, a class of neurons associated with the control of patterned left–right activity (Stokke et al., 2002; Butt and Kiehn, 2003).

Collectively, these findings illustrate that OX can activate spinal circuits at an early age, suggesting a possible role for OX in developing motor circuits. Orexinergic cells are highly activated during walking behavior (Mileykovskiy et al., 2005), and our work illustrates a direct effect of orexin on spinal locomotor networks, suggesting a locus for the locomotor effects of OX.

## Materials and Methods

Experiments were performed on neonatal C57BL/6 mice (age, postnatal day 0 (P0) to P3; *n* = 118) of either sex. All procedures used were approved by the University of Calgary Health Sciences Animal Care Committee (Protocol #AC12-0152 and #AC16-0182) in accordance with guidelines published by the Canadian Council of Animal Care.

### Tissue preparation

#### Spinal cord isolation

Animals were anesthetized by cooling, and decapitated and eviscerated to expose the vertebral column. The remaining tissue was placed ventral side up in a dissection chamber filled with room temperature (RT) oxygenated (95% O_2_, 5% CO_2_) artificial CSF [aCSF (128 mm NaCl, 4 mm KCl, 1.5 mm CaCl_2_, 1 mm MgSO_4_, 0.5 mm Na_2_HPO_4_, 21 mm NaHCO_3_, 30 mm d-glucose)], and spinal cords were exposed via a ventral laminectomy and cutting of dorsal and ventral roots (Whelan et al., 2000). In selected experiments, a slip of ventrolateral funiculus was cut to record from interneuron populations. The spinal cord was removed and left to stabilize for 15–20 min before transferring to a recording chamber, ventral side up, with oxygenated aCSF and gradually heated from room temperature to 27°C. The spinal cord was allowed another 20 min to stabilize before recording commenced.

#### Dorsal horn-removed isolated spinal cord preparation

Dorsal horn-removed isolated spinal cords were prepared using a protocol modified from Dyck and Gosgnach (2009). Spinal cord isolation was performed as described above, with the exception that the dissecting aCSF was kept ice cold (4°C). Following isolation, the spinal cord was transferred to the chamber of a vibratome containing ice-cold, oxygenated (95% O_2_, 5% CO_2_), high-sucrose aCSF (25 mm NaCl, 188 mm sucrose, 1.9 mm KCl, 10 mm MgSO_4_, 1.4 mm Na_2_HPO_4_, 26 mm NaHCO_3_, 25 mm d-glucose). The spinal cord was pinned, dorsal aspect up, along a small valley cut into an agar block, which was fastened to the vibratome specimen plate (VT1000S Vibratome, Leica Biosystems) using cyanoacrylate glue. Serial horizontal sections (100 μm) were removed until the anterior vein was faintly visible through the tissue. The spinal cord was returned to oxygenated, room temperature regular aCSF and was allowed to stabilize for 15–20 min before transfer to the recording chamber. We performed *post hoc* analysis to examine the quantity of dorsal horn tissue removed from L1 to L5 (data not shown).

#### Spinal cord leg-attached preparation

In some experiments we recorded from the tibialis anterior (TA) and triceps surae (TS) muscles. Animals were killed as described above, with dissection occurring in ice-cold, oxygenated (95% O_2_, 5% CO_2_) aCSF (Hayes et al., 2009). Ventral and dorsal laminectomies were performed to the level of S3, with care taken to preserve dorsal and ventral lumbar/sacral roots. The spinal cord was transected at the level of T5–T7 and the dorsal and ventral roots were cut up to T12. The cord was removed from the vertebral column to T13 and non-spinal cord tissue rostral to T12 was discarded. The TA muscle and TS muscle were then exposed through removal of the skin and fasciotomy for electromyogram (EMG) recordings (Pearson et al., 2003). Recordings were done in 27°C aCSF.

### Electrophysiological recordings

Neurograms were obtained from isolated spinal cord experiments by drawing segmental ventral roots from both the right and left ventral roots (L2, one or both left and right ventral roots; L5) into tight-fitting suction electrodes filled with aCSF bath. Suction electrodes were also used to record neurograms from the dorsal L2 and L5 roots in conjunction with the ventral roots for a subset of experiments. Neurograms were amplified (100–10,000 times) and low-pass filtered (1 kHz). In selected experiments, EMG recordings from the TA or TS muscle were obtained using fine silver chloride wires (75 µm; Pearson et al., 2003). EMG recordings were amplified (500 times), and bandpass filtered (100 Hz to 1 kHz). All neurograms or EMG data were digitized (Digidata 1440, Molecular Devices), acquired using Clampex Software (Molecular Devices), and saved on a laboratory computer for off-line analysis.

### Stimulation

Constant current stimulus trains (A360, World Precision Instruments; Master-8 pulse generator, A.M.P.I.) were delivered to the dorsal root (DRL) of L5 through a suction electrode filled with aCSF, and responses were recorded from the ventral lateral funiculi (VLFs) and L5 neurograms. To determine the stimulus-evoked threshold (T), single pulses were delivered at increasing intensities until a polysynaptic reflex response could just be resolved (11-15 μA). Pulses were then delivered every 30 s (stimulus duration, 500 μs; trains, 4 Hz; stimulus intensity, 11-15 μA; 10 pulses, train duration, 10 s) at a constant intensity throughout an experiment to prevent attenuation of the response (Whelan et al., 2000). This was followed by the addition of fast neurotransmission blockers to determine the effects of bath-applied OX acting in OX receptors on MNs and interneurons (VLFs). Single pulses were delivered until no polysynaptic reflex was observed from the VLF and ventral root as a control to determine the efficacy of the fast glutamatergic blockers. Control neurogram activity was recorded continuously for 5 min before OX was bath applied.

### Slice experiments

The isolated spinal cord was prepared as previously described (see Tissue preparation, Spinal cord isolation). Briefly, the spinal cord was dissected free in ice-cold, high-sucrose dissecting solution bubbled with 95% O_2_-5% CO_2_ (25 mm NaCl, 188 mm sucrose, 1.9 mm KCl, 10 mm MgSO_4_, 1.4 mm Na_2_HPO_4_, 26 mm NaHCO_3_, and 25 mm d-glucose). Following dissection of the spinal cord, MNs or descending crossing interneurons (dCINs) were retrogradely labeled by applying crystals of the fluorescent tetramethylrhodamine-conjugated dextran amine [Rhod; molecular weight (MW) 3000; Invitrogen] to the cut ends of their axons in the ventral roots at L1-L2 or to cuts in the spinal cord done at the level of the VLF between the ventral roots L3-L4 using stainless steel pins (Glover, 1995) to retrogradely label MNs and dCINs, respectively (Stokke et al., 2002). Retrograde loading of MN and dCIN somata was allowed to continue at room temperature in the dark for at least 2-3 h in aCSF.

Retrogradely labeled preparations were then transferred in a precooled (4°C) slicing chamber and stabilized in an upright position onto an agar block using 20% gelatin, and the chamber was filled with the ice-cold high-sucrose dissecting solution containing 1 mm kynurenic acid. Transverse sections (250 µm) were cut (Leica VT1000S Vibratome, Leica Biosystems), and the slices were collected in a chamber containing prewarmed (32°C) oxygenated aCSF and equilibrated for at least 45 min before being placed into the recording chamber superfused with oxygenated aCSF solution. The external oxygenated aCSF solution contained 128 mm NaCl, 4 mm KCl, 1.5 mm CaCl_2_, 1 mm MgSO_4_, 0.5 mm Na_2_HPO_4_, 21 mm NaHCO_3_, and 30 mm d-glucose. The internal pipette solution contained 130 mm K gluconate_,_ 0.1 mm EGTA, 10 mm HEPES, 7 mm NaCl, 0.3 mm MgCl_2_, ∼0.4 mm KOH, pH to 7.3, 5 mm di-Tris-creatine, 2 mm ATP (4.8 Tris), and 0.5 mm GTP (1.45 Na^+^). And for some experiments fluorescein isothiocyanate (FITC; 200 μm; MW, 3000; Invitrogen) was added to the intracellular solution (Quinlan et al., 2015) to visualize and localize the recorded cells.

Electrodes were pulled from borosilicate glass on a P97 Flaming/Brown puller (Sutter Instrument) and had resistances in the range of 3–6 MΩ. The liquid junction potential between internal and external solutions was calculated using pClamp 10 Software (Molecular Devices) to be 14.4 mV and corrected.

MNs (soma diameters greater than ∼20 µm) and dCIN soma were visually identified using infrared differential interference contrast (IR-DIC) in the ventral horn of lumbar spinal cord segments 1-2 with a 40× water-immersion objective [LUMPLanFI/IR 40XW, 0.8 numerical aperture (NA), Olympus Canada] of an epifluorescence microscope (BX51WI, Olympus Canada) equipped with a 100 W mercury lamp (TH4-100, Olympus USA) with filters appropriate for visualizing tetramethylrhodamine and fluorescein fluorescence. Labeled cells were identified by the presence of tetramethylrhodamine-conjugated dextran amine-positive somata visualized by fluorescence (excitation, 545/30 nm; emission, 610/75 nm; dichroic filter at 570 nm), and the cells that were intracellularly labeled with fluorescein were also visualized by fluorescence (excitation, 500/24 nm; emission, 542/27 nm, dichroic filter at 520 nm). The data were low-pass filtered (10 kHz) and digitized (sampling rate, 20 kHz) for off-line analysis (Digidata 1440A, Clampex and Clampfit 10, Molecular Devices).

### Data analyses

#### Ventral root neurogram and EMG recordings

Recordings were divided in six segments of 5 min for analysis. The first segment was taken immediately before OX administration, while the next four segments were taken immediately after the addition of OX (total, 20 min). The last 5 min segment was taken after washing the preparation with aCSF for 10 min. Recordings were high-pass filtered (100 Hz), rectified, and smoothed, and the following parameters were measured (using Clampfit 10.4): amplitude, measured from baseline to peak of burst; burst duration, measured from the initiation of the burst to the foot of the burst; frequency, measured by taking the reciprocal of period (duration from the beginning of one burst to the start of the next). To analyze the change in tonic activity induced by bath application of OXs, three segments of 1 min recordings were identified for analysis, and the area under the curve (AUC; in microvolts per millisecond) of filtered, rectified, and smoothed neurograms were obtained. One segment was taken immediately before drug administration, while the next segment was centered around the point where OX had the greatest effect (between 4 and 6 min after addition of OX), and the last one was taken during the washout period. Additionally, for the analysis of fictive locomotor neurograms and EMGs, cross-correlograms between alternating rhythms (right and left L2 and TA, or right L2/L5 and right TA/TS) were also calculated using the rectified, filtered, and smoothed recordings. Data were normalized to control values for each experiment.

#### Effect of orexin on motoneurons and interneurons

For the VLF and MN neurogram recordings, the area under the curve for L5 ventral root neurograms, L3/L4 VLFs, and L5 dorsal root neurograms was taken over the entire period of the experiment to measure the extent of depolarization evoked by OX. Three segments of 1 min recordings were identified for analysis. One segment was taken immediately before drug administration, while the next segment was centered around the point where OX had the greatest effect (between 4 and 6 min after the addition of OX), and the last one was taken during the washout period. Neurograms were filtered and rectified, and the area under the curve was measured. Data were normalized to control values for each experiment.

#### Whole-cell recordings

Only neurons with a resting membrane potential ≤60 mV, an action potential amplitude of at least 60 mV, and a crossing of 0 mV were included in the analysis. A collection of intrinsic electrical properties was recorded from each cell (total MNs, *n* = 22; dCINs, *n* = 14) in this study. These properties were as follows: action potential amplitude, action potential threshold voltage, membrane capacitance (Cm), rheobase current, input resistance (R_in_), capacitance, and frequency*–*current slope (*F–I* gain). To assure uniformity of the measurements, all neurons were held below threshold at −75 mV with a bias current. Action potential amplitude was measured from the resting potential preceding an action potential to that action potential peak. Rheobase current was quantified as the minimal depolarizing current step (2 Hz, 25 ms duration) sufficient to elicit an action potential in our measurements. R_in_ was estimated by dividing the voltage deflection of the membrane potential by hyperpolarizing current pulses. Whole-cell Cm (in picofarads) was recorded using the automated membrane test function in Clampex 10 (Molecular Devices); briefly, in voltage-clamp mode, after achieving the whole-cell configuration, a 10 mV command pulse is delivered and the resulting estimated integral of the current transient relative to the steady-state current during the pulse plus a steady-state correction factor are used to calculate the whole-cell capacitance. To determine the *F–I* plot, 0.5 s current injections of increasing amplitude were delivered, and the average spike frequency was determined by counting the number of spikes during the 0.5 s step, and the instantaneous frequency was defined and measured as the first interspike interval during a step; these were plotted against the injected current amplitude. The slope of the *F–I* curve was linearly fitted. In all cases when the membrane potential (V_m_) was depolarized by OXs, we injected hyperpolarizing bias current to return the V_m_ to predrug levels.

### Immunohistochemistry

#### *Post hoc* patched neurons

Cell morphology, as revealed by the fluorescent dye fill, was acquired at the end of the electrophysiological recordings (analysis of images, Pinnacle Studio 12, Corel). The section (250 µm) containing the patched cells filled with the intracellular solution containing fluorescein dextran amine were then put overnight in a 96-well plate with 4% paraformaldehyde at 4°C (some sections with no recorded/filled cells were kept to be used as control slices for the subsequent immunohistochemistry). The sections were washed in 1× PBS (154 mm NaCl, 7.7 mm Na_2_HPO_4_, 2.7 mm NaH_2_PO_4_ in double-distilled H_2_O), followed by a blocking and permeabilization step in a solution consisting of 10% normal donkey serum in 0.5% PBST (0.5% Triton X-100/1× PBS) for 6 h at RT (∼22–25°C) on a shaker. The same blocking solution was used in all subsequent antibody incubations. The sections were then incubated in primary antibody solution overnight at RT on a shaker. For the sections containing the patched cells they were filled with the intracellular solution containing FITC and MNs or dCINs retrogradely labeled with the Rhod, primary antibody solution, 1:1000 goat α-FITC/Oregon Green (A-11095, lot #1485201, Thermo Fisher Scientific), and 1:1000 rabbit α-tetramethylrhodamine, polyclonal IgG (A-6397, lot #1476653, Thermo Fisher Scientific). MNs that were not previously backfilled with rhodamine were also intracellularly filled with FITC (and the cholinergic nature of those cells was verified), 1:1000 goat α-ChAT (AB_144_P, lot #2558394, EMD Millipore), and 1:1000 rabbit α-FITC/Oregon Green-488 (A-11090, lot #1571691, Thermo Fisher Scientific). Sections were washed in 0.5% PBST, and the incubation with the secondary antibody solution was performed for 6 h at RT. The appropriate secondary antibody solution contain 1:1000 Alexa Fluor 488 donkey α-rabbit (A-21206, lot #1182675, Thermo Fisher Scientific) or 1:1000 donkey α-goat (A-11055, lot #1627966, Thermo Fisher Scientific) and 1:1000 Alexa Fluor 594 donkey α-rabbit, polyclonal IgG (A-21207, lot #1668652, Thermo Fisher Scientific) or Alexa Fluor 568 donkey α-goat 1:1000 (A-11057, lot #1640316, Thermo Fisher Scientific). The sections were then washed with 1× PBS, mounted onto Superfrost Plus slides (VWR International) under an epifluorescent microscope to ensure that the filled cells are on the appropriate side of the slide to face the microscope lens, mounted with Fluoromount/Plus medium (Diagnostic Biosystems), coverslipped, and sealed with clear nail polish.

#### Orexin fibers

Spinal cords were removed as previously described (Glover, 1995) and transected at T11/T12, and tetramethylrhodamine-conjugated dextran amine was applied to the ventral roots of the neonatal spinal cord (right and left from T13/L1 to L5; compare with Slice experiments) . Spinal cords were then submerged in 10% formalin at 4°C overnight, then cryoprotected in 30% sucrose/1× PBS for 24–48 h at 4°C. The spinal cords were embedded in clear frozen section compound (OCT, VWR International). The 30 μm longitudinal and transverse spinal cord sections were cut using a cryostat (Leica CM1850 UV, Leica Biosystems). The spinal cord sections were mounted onto Superfrost Plus Slides (VWR International). All slides were dried overnight at RT, then rehydrated in 1× PBS for 5 min and washed three times in 0.3% PBST (0.3% Triton X-100 in 1× PBS) for 10 min each. Tissue sections were encircled with a hydrophobic border using a PAP Pen (Daido Sangyo) to contain and conserve solutions. A blocking solution containing 0.3% PBST and 5% normal donkey serum was then added for 1 h. The same blocking solution was used as the antibody diluent in subsequent steps. Primary antibody solution [1:2000 rabbit α-OXA, polyclonal IgG (AB3704, lot #2887990, Millipore Sigma] was added, and slides were incubated overnight (∼10 h). The next day, tissue sections were again washed three times in 0.3% PBST (10 min), then incubated for 1 h in secondary antibody solution (1:1000 Alexa Fluor 488 donkey α-rabbit, polyclonal IgG; A21206, lot #1531671, Millipore Sigma). Tissue was then washed a final three times in 1× PBS for 10 min each. The hydrophobic barrier was carefully wiped away, and tissue was mounted in Vectashield Mounting Medium (H-1000, Vector Laboratories) under a coverslip, and sealed at the edges with clear nail polish. Residual salts were cleaned off the slides with a wet (distilled H_2_O) Kimwipe. All washes and incubations were conducted at RT in a humidified box. To test for nonspecific binding of the secondary antibodies, controls were set up in each experiment that excluded the primary antibodies, and lateral hypothalamic brain slices from adult mice were stained for OXergic cells as a positive control.

### Imaging

Stitched fluorescence images were collected using an Olympus VS120 Virtual Slide Scanner (Olympus Canada). The objective used was UPlanSApo 20×/0.75 air, illuminated by an X-Cite Exacte Mercury Vapor Short-Arc Lamp. The filters used were green (excitation, 485/20; emission, 525/30) and red (excitation, 560/25; emission, 607/36). Images were acquired as a *Z*-stack at 1376 × 1038 pixel resolution. Off-line image processing included EFI processing using cellSens Dimension Desktop 1.12 (Olympus Canada) as well as adjustments of brightness and contrast in Photoshop CS6 13.0.1 (Adobe Systems). Confocal fluorescent images were collected using a Nikon A1R Multiphoton Microscope (Nikon Canada). The objective used was 60× Plan Apo water-immersion (NA, 1.27). The laser was centered on 488 nm (with a 525/50 emission filter) and 561 nm (with a 600/50 emission filter) wavelengths. Images were acquired as a *Z*-stack with 2.3 μs pixel dwell, 2× line averaging, and 1024 × 1024 pixel resolution. Off-line image processing included maximal intensity projections conducted using NIS-Elements Advanced Research version 4.10 (Nikon Canada) as well as adjustments of brightness and contrast in Adobe Photoshop. The same adjustments and settings were used for all images within a given series for image consistency and comparison.

### Pharmacology

Fictive locomotion was elicited in isolated spinal cord and intact hind-limb preparations by bath application of NMDA (5 µM; Sigma-Aldrich), 5-hydroxytryptamine (5-HT, 10 µM; Sigma-Aldrich), and dopamine (DA; 50 μm; Sigma-Aldrich) made fresh daily. OX receptors were targeted using OXA or OXB (300 nm; Tocris Bioscience) as an agonist. A dual OX receptor (OXR) antagonist, TCS 1102 (10 μm; Tocris Bioscience) was used in conjunction with OXA or OXB (300 nm) as a control. To derive the effect of OX on network-isolated interneurons and MNs, fast neurotransmission blockers were used. Stock solutions (10–100 mm) of fast neurotransmission blockers were kept at −20°C until needed. During the experiment, all drugs were dissolved in regular aCSF, composed of the following: 6,7-dinitroquinoxaline-2,3-dione (DNQX; AMPA/kainate receptor antagonist, 20 μm; Tocris Bioscience), picrotoxin (PTX; GABA_A_ receptor antagonist, 50 μm; Sigma-Aldrich), strychnine (glycine receptor antagonist, 10 μm; Sigma-Aldrich), dl-2-amino-7-phosphonovalerate (dl-APV; NMDA receptor antagonist, 50 μm; Tocris Bioscience), and mecamylamine hydrochloride (nicotinic acetylcholine receptor antagonist, 100 μm; Tocris Bioscience).

### Statistics

The mean values of each variable obtained during the control condition [pre-OX (before drug application)] compared with those obtained after drug application (orexin) and washout of the drug were compared using a nonparametric repeated-measures ANOVA (Friedman test). All statistics were performed on non-normalized data. *Post hoc* analysis was conducted when significant effects were detected with ANOVAs. Paired and unpaired *t* tests were conducted for between-group comparisons of two groups using GraphPad Prism 6.0 (GraphPad Software). The level of significance was set at *p* < 0.05 for all conditions. Data were reported as the mean ± SD.

## Results

### Orexin fibers are present in the lumbar spinal cord at early stages

**Figure 1.**
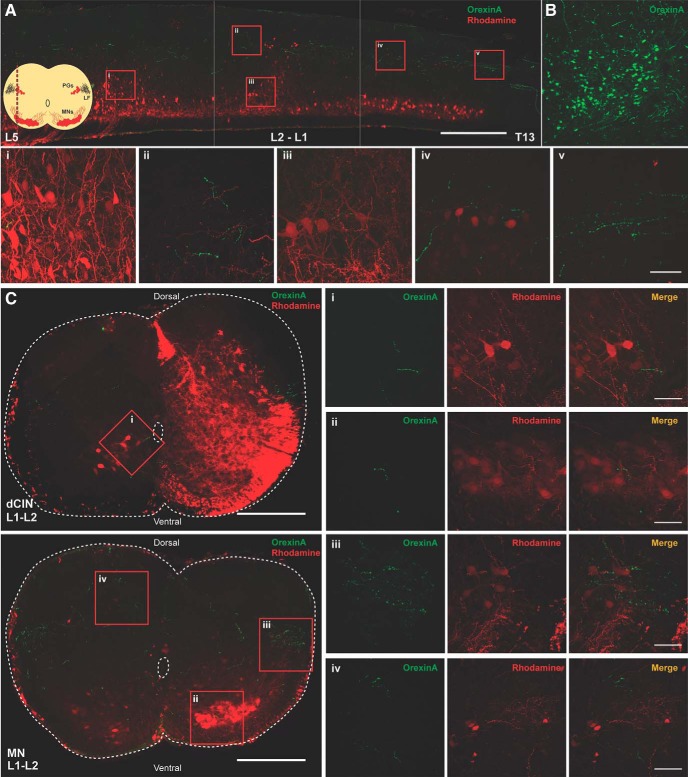
Orexinergic innervation of the developing (P3) spinal cord. ***A***, Longitudinal section of spinal cord (30 μm) showing numerous orexinergic fibers projecting along the lateral funiculus throughout the lumbar spinal cord. Scale bar, 500 µm. Corner schematic shows the location of the longitudinal section (LF, lateral funiculus; PG, preganglionic motor neurons); 60× confocal images of select regions (scale bar, 50 µm): ***i***, sparse orexinergic fibers around MNs in lumbar region; ***ii***, dorsal fibers in the L1-L2 region; ***iii***, rare fiber passing by L1-L2 MNs; ***iv***, orexinergic fibers exiting the LF around the preganglionic MNs; ***v***, orexinergic fibers in the LF, varicosities are evident. ***B***, Positive control showing OXergic neurons in lateral hypothalamus. ***C***, Transverse sections of L1-L2 spinal cord with rhodamine-labeled dCINs and rhodamine-labeled ventral MNs (scale bar, 250 µm); 60× confocal images of select regions (scale bar, 50 µm): ***i***, orexinergic fibers apposing dCINs; ***ii***, sparse fibers in MN pool; ***iii***, orexinergic fibers exiting the LF around the preganglionic MNs; ***iv***, dorsal fibers are prevalent.

We first determined whether OX-IR^+^ fibers were present in the spinal cord perinatally. Using both transverse and longitudinal sections, we examined expression within the low thoracolumbar spinal cord (T13–L6). We found that OX-IR^+^ could be observed in the lateral white matter, along the lateral funiculus of the spinal cord and within the spinal cord (P3; Fig. 1*A*, *v*, *C*, *iii*). Fibers could be detected in all laminae of the spinal cord, including the ventral horn (Fig. 1*C*), but primarily in the dorsal laminae (Fig. 1*A*, *ii*, *C*, *iv*). In sections where we retrogradely labeled MNs with tetramethylrhodamine-conjugated dextran amine, OX-IR^+^ fibers abutting MN dendrites could be visualized using longitudinal and transverse slices (Fig. 1*A*, *i*,*iii*, *C*, *iii*). In L1-L2 segments, we could observe preganglionic neurons that were also retrogradely labeled, and again fibers were found in proximity to these cells (Fig. 1*A*, *iv*, *C*, *iii*). Some fibers were observed in lamina VIII close to dCINs retrogradely loaded with tetramethylrhodamine-conjugated dextran amine (Fig. 1*C*, *i*). Positive controls were performed by using adult lateral hypothalamus sections where the robust staining of cells was observed as previously published (de Lecea et al., 1998; Sakurai et al., 1998; van den Pol, 1999; Fig. 1*B*)**.**

### Effect of orexin A and B on spontaneous activity

**Figure 2.**
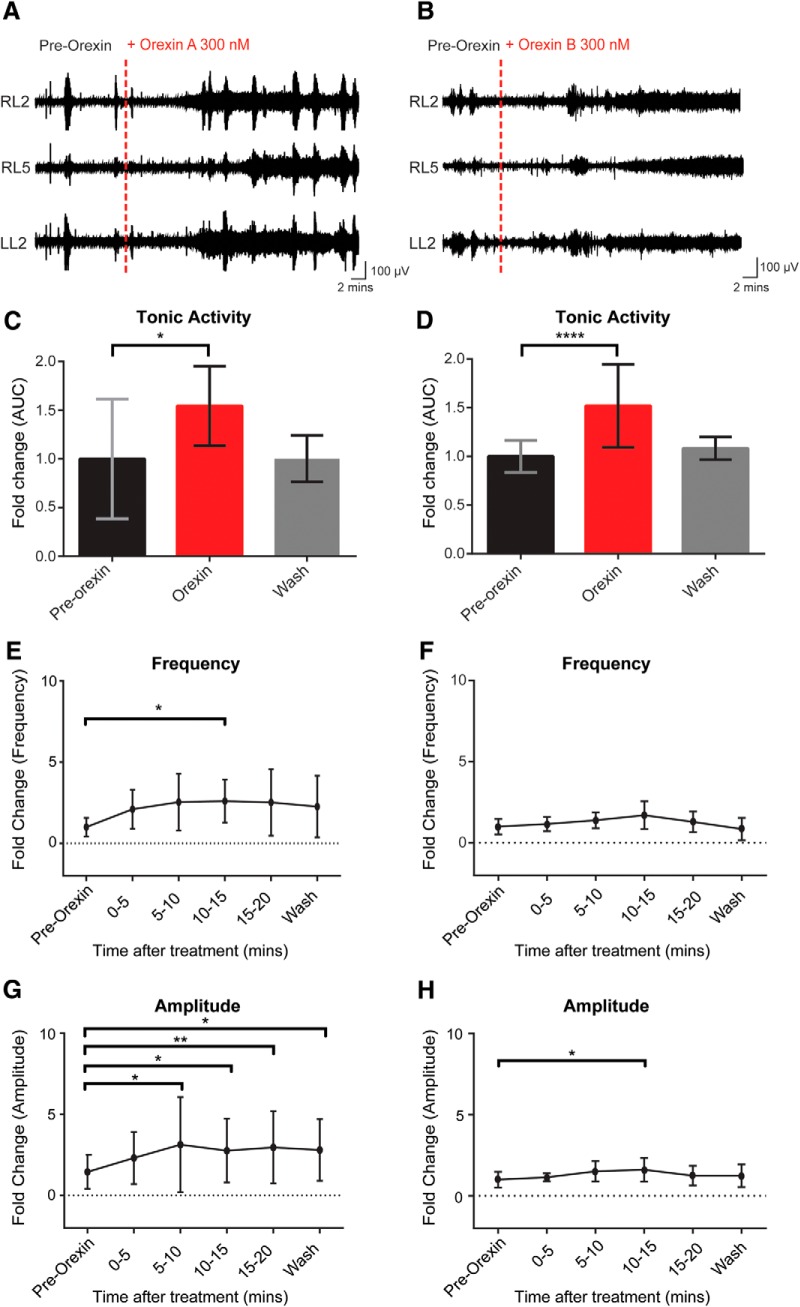
Effect of OXA and OXB on spontaneous spinal cord motor activity. ***A***, ***B***, Representative neurogram of recorded motor spontaneous activity in absence of any drugs (pre-OX) and after bath application of OXA (***A***) and OXB (***B***; OXA and OXB, 300 nm, were added at the red dotted lines). ***C***, ***D***, Bar graphs representing the fold change of the tonic activity calculated as the AUC in different conditions: pre-OX (black); OXA (***C***) and OXB (***D***; 300 nM; red); washout of OXA or OXB (gray). ***E***, ***F***, Graphs representing the time course of the fold change in frequency of the spontaneous activity comparing pre-OX with the frequency observed at different times after OXA (***E***) and OXB (***F***) bath application. ***G***, ***H***, The fold change in amplitude of the spontaneous bursts during OXA and OXB bath application, respectively. *N* = 7/group for OXA and *N* = 10/group for OXB. RL, Right lumbar ventral roots; LL, left lumbar ventral roots (recorded at the second or fifth lumbar segmental level). Data are represented as the mean ± SD. *p < 0.05, **p < 0.01, ****p < 0.0001.

Spontaneous activity is a feature of developing systems and has been shown to affect synaptic connectivity in motor networks (Blankenship and Feller, 2010; Jean-Xavier et al., 2018). Since it is an endogenous activity that can be recorded without pharmacological stimulation, it provides a useful approach for examining the efficacy of neuromodulators. Therefore, we investigated the effects of OXA and OXB at 300 nm on spontaneous motor network output. Bath application of OXA to the isolated *in vitro* spinal cord preparation led to a significant increase in the discharge recorded from L2 and L5 ventral roots (Fig. 2*A*,*B*). Tonic activity depicts an increase in the overall firing rate of the flexor-related (L2) and extensor-related (L5) neurograms, while frequency measures the change in the number of bursts (Fig. 2). All parameters for each experiment were analyzed for different segmental levels, mainly for L2 and L5 ventral roots; similar changes were observed for all parameters for all experiments for both L2 and L5 ventral roots. For clarity purposes, we report that the statistical analysis for L2 in the absence of differences between segments. Analysis of the data indicated that OXA led to an increase in the tonic activity as well as burst episodes of the neurograms (*N* = 7; mean fold change tonic activity = 1.54 ± 0.41; *p* = 0.015; Fig. 2*C*). Comparing the frequency (Fig. 2*E*) and amplitude (Fig. 2*G*) in control condition (pre-OXA application) with the frequency and amplitude observed at different times following the bath application of OXA, we observed that it takes at least 5 min for OXA to have a statistically significant effect on these parameters (*N* = 7; mean fold of change frequency = 2.54 ± 1.75, *p* = 0.022; mean fold of change amplitude = 2.62 ± 2.45, *p* = 0.022; Fig. 2*C*,*E*,*G*). However, there was no significant change in burst duration (*p* = 0.77; data not shown). Similar to OXA, the addition of OXB led to an increase in the tonic activity (Fig. 2*B*) but did not significantly affect the frequency (Fig. 2*F*) of the spontaneous bursts (*N* = 10; mean fold change tonic activity = 1.52 ± 0.43, *p* < 0.0001; frequency, *p* = 0.94; Fig. 2*D*,*F*). The spontaneous burst amplitude after 10 min of OXB application was significantly increased compared with pre-OXB application (mean fold of change amplitude = 1.61 ± 0.72; *p* = 0.03) even if the effect of OXB on the burst amplitude was less prominent than after OXA application (Fig. 2*H*). These results indicate that both OXA and OXB are capable of increasing ventral root neurogram discharge. In addition, OXA was also capable of increasing spontaneous bursting frequency. Our data suggest that OXA, but not OXB, can modulate the network controlling the generation of spontaneous episodes of activity. Thus, at early ages, OXR activation via OXA and OXB ligands is capable of distinct activation of lumbar networks.

### Orexin receptor antagonist abolishes the effect of orexin

**Figure 3.**
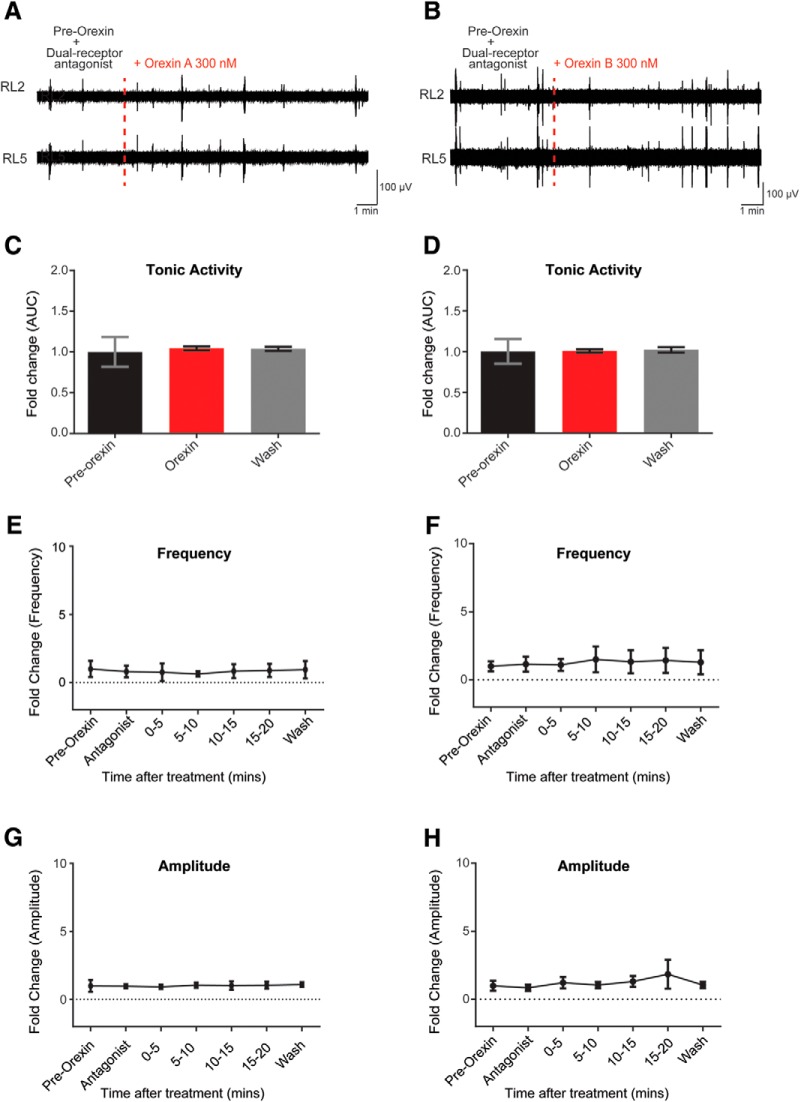
OXR antagonist abolished OXA or OXB-mediated excitation. ***A***, ***B***, Representative neurogram of recorded motor spontaneous activity when the OX1R and OX2R (dual receptor) antagonist TCS 1102 (10 μm) was applied to the isolated spinal cord preparation before the addition of 300 nm OXA (***A***) or OXB (***B***; indicated at the red dotted lines), respectively. Pre-OX + antagonist indicates period when antagonist was added to the aCSF. ***C***, ***D***, Bar graphs representing the fold change of the tonic activity: pre-OX + antagonist (black); OXA (***C***; 300 nm; red); OXB (***D***; 300 nm); washout (gray) of OXA (***C***) or OXB (***D***) in the presence of dual antagonist. ***E***, ***F***, Graphs representing the evolution of the fold change in frequency of the spontaneous activity comparing pre-OX, OXA (***E***), or OXB (***F***) observed at different times following the application of OXA or OXB in the presence of TCS 1102. ***G***, ***H***, The fold change in amplitude of the spontaneous bursts during OXA or OXB bath application, in the presence of TCS 1102. *N* = 4 for the dual receptor antagonist experiments, respectively. Data are represented as the mean ± SD.

To determine whether the effects of OXA and OXB were on OX1 and OX2 receptors, we used OX receptor antagonists to block receptor function. OXA has equal affinity for both OX1 and OX2 receptors (Sakurai et al., 1998). We bath applied OXA in the presence of a dual OX1 and OX2 receptor antagonist (TCS 1102, 10 μm) and found that visible increases in discharge in the ventral root neurograms were abolished (*N* = 4; mean fold change tonic activity = 1.04 ± 0.024, *p* = 0.44; frequency, *p* = 0.70; amplitude, *p* = 0.99; Fig. 3*A*,*C*,*E*,*G*). The effects of the bath application of OXB were also blocked with the addition of the dual receptor antagonist (*N* = 4; mean fold change tonic activity = 1.01 ± 0.021, *p* = 0.53; frequency, *p* = 0.18; amplitude, *p* = 0.40; Fig. 3*B*,*D*,*F*,*H*). In addition, those experiments allowed us to test the contribution of endogenous OXs. The addition of the OX1/2 antagonists did not affect any parameters of the spontaneous activity recorded and analyzed (*p* = 0.13), suggesting that endogenous release of OXs binding to OX1/2 receptors was not a major factor and that there is no significant effect of constitutively active OX receptors. This was done by analyzing the spontaneous activity parameters in control conditions (regular aCSF; pre-OX; Fig. 3), with the activity recorded with the receptor antagonists added to the aCSF (Fig. 3).

### Effect of orexin A and B on fictive locomotion

**Figure 4.**
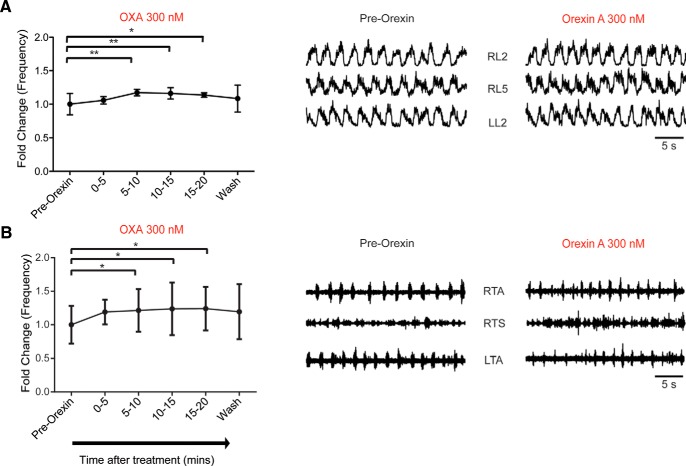
Orexin A modulates fictive locomotor rhythm frequency. ***A***, Graph representing the time course of change in the frequency of fictive locomotor rhythm comparing the pre-OX frequency with the one observed at different times after the bath application of OXA (300 nm) obtained from an isolated spinal cord preparation (*N* = 7). Right, Representative neurograms showing the increase in frequency of the fictive locomotor rhythm induced by bath application of DA, NMDA, and 5-HT before and during OXA bath application. ***B***, Graph representing the time course of the change in frequency of fictive the locomotor rhythm comparing pre-OX frequency with the one observed at different times after the application of OXA (300 nm) obtained from a spinal cord with leg-attached preparation (*N* = 5 preparations). Right, Representative EMG recordings showing the increased frequency of the fictive locomotor rhythm induced by bath application of DA, NMDA, and 5-HT before and during OXA bath application. RL, Right lumbar ventral roots; LL, left lumbar ventral roots (recorded at the second or fifth lumbar segmental level); RTA, right TA muscle; LTA, left TA muscle; RTS, right TS muscle. Data are represented as the mean ± SD. *p < 0.05, **p < 0.01.

Given that OXs can modulate spontaneous activity, we next sought to examine whether the OXergic system could also modulate pharmacologically induced fictive locomotion. Fictive locomotion is produced by a central pattern generator (CPG) in the spinal cord, which has been extensively characterized (Kiehn, 2016). Fictive locomotion was induced by bath application of DA, NMDA, and 5-HT, and was then followed by bath application of OXA. As early as 5 min following the application of OXA, there was a significant increase in fictive locomotor rhythm frequency (*p* < 0.05). However, there were no significant differences between control (pre-OX application) and OXA for cross-correlation value between left/right L2 and between L2 and L5 ventral roots, or amplitude and burst duration (*N* = 7; *p* > 0.05; data not shown). The frequency change observed following OXA application peaked at 5–10 min after the addition of OXA (*N* = 7; mean fold change, 1.17 ± 0.048, *p* = 0.0032; Fig. 4*A*). This effect lasted for the duration of the OXA bath application and was abolished within 10 min after washing out OXA with aCSF (plus fictive locomotion-inducing drugs). The reduction in the effect of OXA after 10 min may reflect the desensitization of OX1 G-protein-coupled receptors. OXB did not have an effect on spinal locomotor frequency at concentrations similar to that of OXA (300 nm; *N* = 5; frequency, *p* = 0.96; data not shown). We performed a set of control experiments where we monitored rhythm stability following DA, NMDA, and 5-HT application over time without bath application of OX agonists or antagonists. There was no difference in any of the analysis parameters in these experiments, which suggested that the effects of OXA were not simply due to changes in network function over time (*N* = 5; frequency, *p* = 0.68; data not shown).

Another possibility tested was whether the changes in fictive locomotor frequency were due to the effects of OXA on recurrent collaterals from MNs, a pathway reported to be important for the genesis of rhythmic activity (O’Donovan et al., 2010; Falgairolle et al., 2017). We blocked cholinergic inputs from recurrent collaterals following the administration of 5-HT/NMDA/DA to evoke locomotor activity by bath applying 100 µm mecamylamine. OXA was capable of increasing the frequency of fictive locomotor activity in the L2 neurograms (*N* = 7; *p* = 0.0004). The effect was most pronounced during the 5–10 and 10–15 min intervals post-orexin application when compared to the 5 min pre-orexin period (mean fold change in frequency in the 5–10 min interval: 1.17 ± 0.16, *p* = 0.0033; mean fold change in frequency in the 10–15 min interval: 1.16 ± 0.17, *p* = 0.0020; data not shown). These data do not rule out a glutamatergic contribution from the recurrent collaterals (Mentis et al., 2005; Nishimaru et al., 2005).

To resolve whether orexin was producing a more discrete change in flexor–extensor coordination we used a spinal cord with leg-attached preparation following the addition of OXA (Pearson et al., 2003). This leg-attached preparation also indicates the effect of orexin on motor output independent of direct effects on preganglionic neurons (L2 neurograms). The data show an increase in frequency following the bath application of OXA for the TA and the TS muscles (*N* = 5; mean fold change: 1.39 ± 0.23, *p* = 0.020), similar to the isolated spinal cord preparation (Fig. 4*B*). One difference is that the effects appeared to be sustained and did not washout. We did not observe any changes in the patterning of locomotion.

Altogether, these data suggest that OXA and not OXB is able to modulate the frequency of the fictive locomotor rhythm and that recurrent collaterals from MNs, at least via their cholinergic component, are not involved in this change in network function.

### Effect of orexin on motoneurons and interneurons

We next tried to identify the cellular target of OXs. We used suction electrode recording techniques in conjunction with the bath application of fast neurotransmission blockers (DNQX, PTX, strychnine, and dl-APV) to capture the effects of OX on synaptically isolated populations of neurons. The populations of interest were MN and interneuron populations, whose signals were recorded via neurograms from a given ventral root as well as activity from ventral horn interneurons projecting into the VLF, respectively. The VLF contains neurons that project from ipsilateral segments and from contralateral commissural interneurons (Stokke et al., 2002). A caveat is that it does not reflect local interneuronal activity. This approach allows for isolating the effects of OXA on the MNs and selected interneurons in the spinal cord with a minimal contribution from the network.

**Figure 5.**
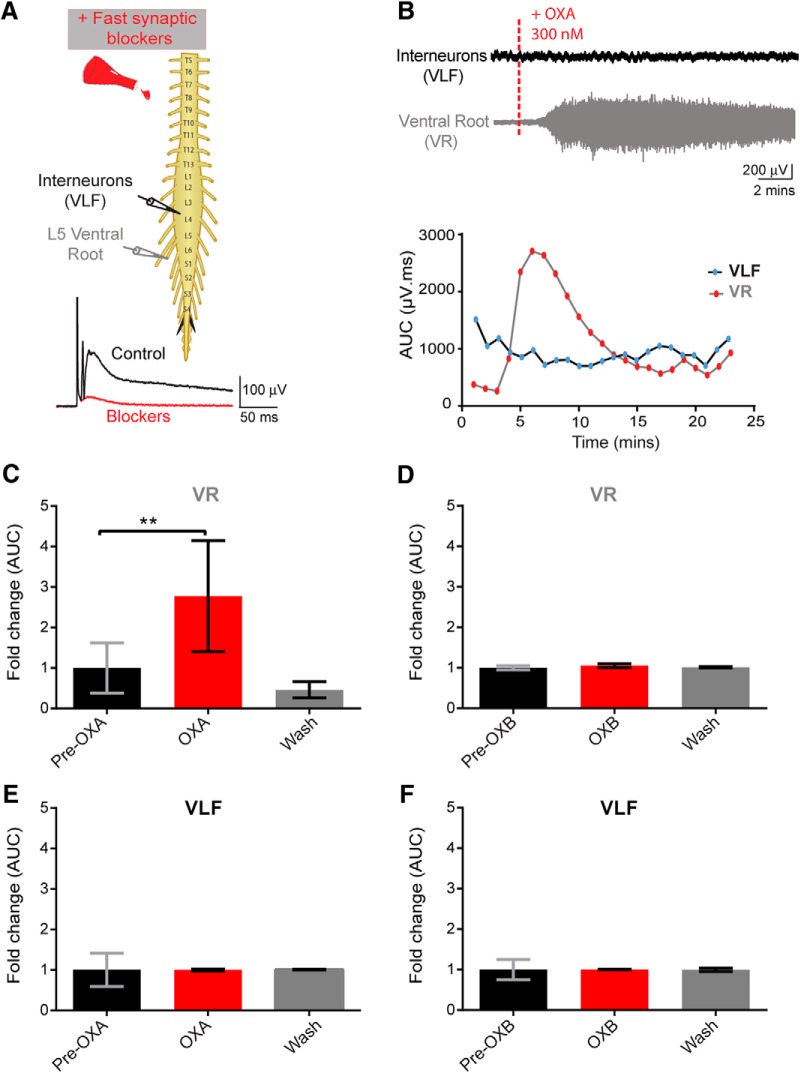
Orexins effects on motoneuron and interneuron populations. ***A***, Top, Experimental setup; pulses delivered every 30 s were delivered to the L5 dorsal root through a suction electrode to elicit an evoked response recorded via suction electrodes placed on the right L5 ventral root (VR, recording of motoneuron pool) and onto the right L4 or L3 VLF to record activity from ventral horn interneurons projecting into the VLF. Bottom, Following the addition of fast neurotransmitter blockers, single pulses were delivered, and an evoked polysynaptic response was recorded from the VR and VLF (control, black trace) until no polysynaptic reflex was present (blockers, red trace). ***B***, Top, Representative neurograms showing that the bath application of OXA 300 nm induced an increase in neurogram discharge recorded from the VR but not from the VLF. Bottom, Graphs showing the analysis of the change in tonic activity induced following bath application of OXA, a minute-by-minute analysis of the AUC (in microvolts per millisecond) of filtered, rectified, and reduced neurograms were obtained. ***C***, ***E***, Graphs representing the time course of the fold change in tonic activity from VR (***C***) and VLF (***E***) neurograms comparing the pre-OXA and post-OXA AUC (300 nm; *N* = 6 preparations). ***D***, ***F***, Graphs for pre-OXB and post-OXB for the VR (***D***) and VLF (***F***), respectively. Data are represented as the mean ± SD. **p < 0.01.

As early as 5 min after the bath application of OXA, there was a significant increase in the ventral root discharge (*N* = 6; mean fold change: 2.77 ± 0.56, *p* = 0.0001; Fig. 5*B*,*C*). Furthermore, washing off OXA with aCSF containing fast neurotransmission blockers decreased the tonic discharge recorded from ventral root neurograms (Fig. 5*B*,*C*). We never observed that OX application evoked rhythmic activity under these conditions. No effect of OXA was observed on VLF neurograms (interneurons) in the presence of fast neurotransmission blockers (*N* = 6; *p* = 0.14; Fig. 5*B*,*E*). To confirm that the VLF preparation was viable and the lack of effect of OXA on VLF neurogram was accurate, we assessed the effect of DA on the same VLF spinal cord preparation. DA has been shown to increase firing in the VLF (Han et al., 2007). Administering DA following washout of OXA with aCSF (containing DNQX, PTX, strychnine, and dl-APV) led to a clear discharge of the VLF neurogram (data not shown). These results suggest that the lack of a response from the VLF neurogram was not due to the viability of the preparation or an artifact of the preparation/recording methods, but instead is due to lack of an observable effect of OXA on the VLF interneuron population.

We next investigated the effect of OXB on isolated spinal cord preparations with fast neurotransmission blockers (similar conditions as for OXA). Surprisingly, the bath application of OXB did not produce any discernible changes on the neurogram discharge from spinal cord MNs (*N* = 5; *p* = 0.37; Fig. 5*D*) and from ventral horn interneurons projecting into the VLF (*N* = 5; *p* = 0.95; Fig. 5*F*). These results suggest that OXB does not have an observable effect on spike activity from the two populations of neurons sampled, at least with fast synaptic blockers on board.

**Figure 6.**
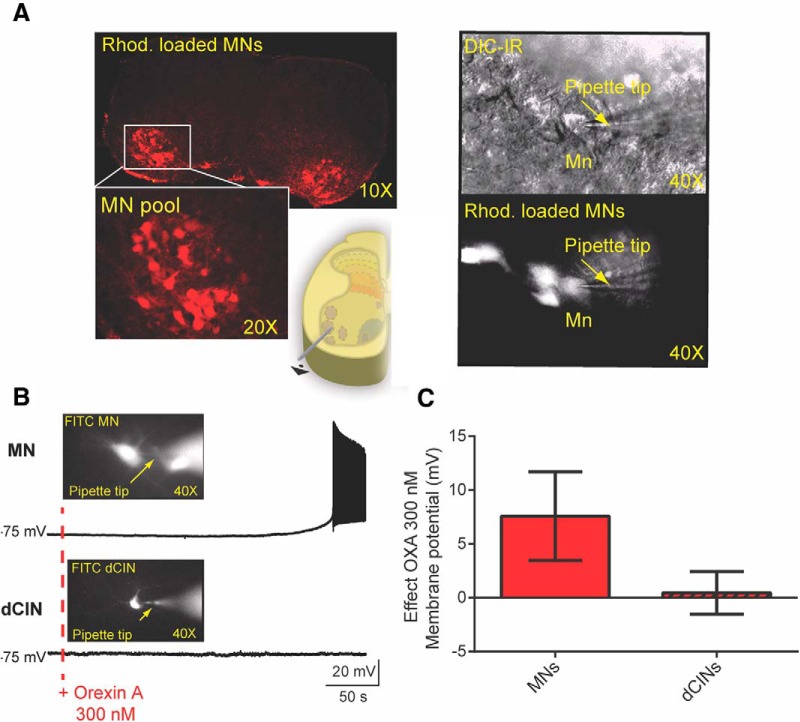
The effect of orexins on identified MNs and interneurons. ***A***, Left, A 10× picture of a spinal cord showing MN pools previously retrogradely labeled by fluorescent Rhod crystals applied to the cut ends of ventral root L1-L2 axons. Bottom, A higher magnification of the MN pool (20×) and the schematic illustrating the ventrolateral position of the MN pools. Right, MN visually identified using IR-DIC optics in the ventral horn of the L1-L2 transverse slice (bottom right). ***B***, OXA led to a depolarization of the MN recorded, but not the dCINs. The pictures show the patched cells that were visually identified via Rhod. Retrograde labeling, and the cells were also intracellularly labeled with FITC to identify *post hoc* which cells in the pool were recorded. ***C***, Quantification of the effects of OXA 300 nm on MNs and dCINs (MNs, *N* = 22; dCINs, *N* = 14).

**Figure 7.**
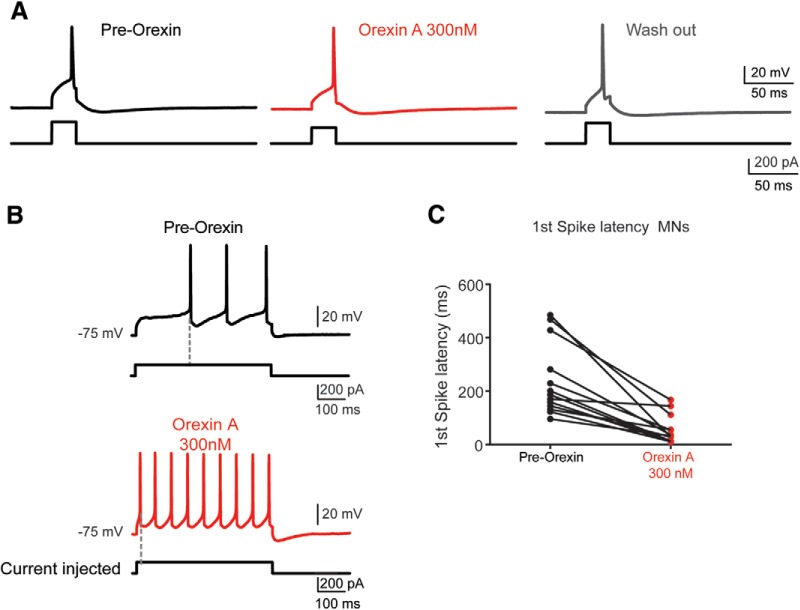
Orexin A modulates some intrinsic properties of identified motoneurons. ***A***, Representative example of intracellular recordings showing an increase in the MN rheobase following OXA 300 nm (red traces). ***B***, Bath application of OXA 300 nm blocked the delay of firing in an identified L1/2 MN. There is a shift toward the left for the spiking (see dashed line for the same MN and same current injected). ***C***, Summary plot of changes in the first spike latency pre-OX and post-OX (MNs, *N* = 13). See Tables 1 and 2 for further details.

As OXA was more potent than OXB in acting on synaptically isolated cell populations (Fig. 5), we next sought to determine the effect of OXA on intrinsic cellular properties. Using lumbar transverse slice preparations, we obtained a whole-cell patch recording from MNs and interneurons identified by size and by retrograde labeling with fluorescent tetramethylrhodamine-conjugated dextran amine (Fig. 6*A*,*B*). Our data show that OXA led to a depolarization of all MNs recorded (7.58 ± 4.11 mV; *N* = 22; Fig. 6*C*), similar to what we observed using extracellular recordings from ventral roots in the presence of fast neurotransmission blockers. Supporting this finding, we found that the R_in_ was significantly increased. The rheobase was significantly decreased, and, somewhat surprisingly, the gain as indicated by the average slope was decreased (Table 1, details including statistics; Fig. 7*A–C*). When we examined the latency to the first spike following a current step, we found that the latency was significantly shortened, suggesting an effect on transient outward rectifying A type (I_A_) conductances (*N* = 13 MNs; 238.6 ± 135.6–54.1 ± 53.0 ms; Fig. 7*B*,*C*; Shibata et al., 2000; Russier et al., 2003). Collectively, these data suggest that MNs following bath application of OXA could be recruited using lower injected current compared to pre-OXA conditions.

**Table 1:** Summary of orexin A effects on some intrinsic properties recorded from motoneurons

	MNs		
Parameters/conditions	Pre-OX	OXA 300 nm	Washout
R_in_ (MΩ)	80.60 ± 46.02 (13)	102.43 ± 38.41 (13)**	92.16 ± 43.04 (11)^ns^
AP amplitude (mV)	72.48 ± 8.89 (13)	68.59 ± 10.61 (13)^ns^	69.08 ± 10.54 (12)^ns^
AP threshold (mV)	−51.08 ± 8.90 (13)	−51.32 ± 8.87 (13)^ns^	−52.34 ± 9.90 (12)^ns^
Rheobase (pA)	478.6 ± 211.4 (13)	396.7 ± 181.7 (13)*	433.1 ± 233.3 (11)^ns^
Average *F*/*I* slope (pA/Hz)	0.0855 ± 0.0692 (13)	0.0699 ± 0.0451 (13)*	0.0705 ± 0.0284 (10)^ns^
Instantaneous *F*/*I* slope (pA/Hz)	0.1786 ± 0.206 (13)	0.1468 ± 0.165 (13)^ns^	0.1505 ± 0.209 (10)^ns^

Values are the mean ± SD (number of motoneurons). Report of the statistical significance between control condition, pre-OX and OXA at 300 nm, and the control and washout of OXA for the following parameters: AP amplitude; action potential amplitude, AP threshold, action potential threshold, rheobase; average *F*/*I* slope, average frequency of spiking/intensity of current-injected slope; instantaneous *F*/*I* slope, instantaneous frequency/intensity slope. One-way repeated-measures ANOVA followed by Student–Newman–Keuls *post hoc* test was used to determine the significance between conditions.

^ns^*p* > 0.05; *p ≤ 0.05; **p ≤ 0.01.

OXA may still have subthreshold effects on VLF interneurons not reflected by extracellular recording techniques. One population that projects to the VLF, and which also could be expected to affect locomotor frequency, is the dCIN population (Stokke et al., 2002; Butt and Kiehn, 2003). So, to tackle this issue, we retrogradely labeled VLF projecting neurons and deployed whole-cell patch approaches to record from visually identified dCINs (Eide et al., 1999; Butt et al., 2002). Our data show that OXA did not depolarize the dCINs recorded (*N* = 14 dCINs; 1.22 ± 2.98 mV; Fig. 6*C*), confirming what we observed using extracellular recordings from VLF in the presence of fast neurotransmission blockers. We found that OXA bath application had no significant effect on a variety of intrinsic properties of these dCINs (Table 2, Fig. 6*B*,*C*).

**Table 2:** Orexin A does not affect the intrinsic properties recorded from dCINs

	dCINs		
Parameters/conditions	Pre-OX	OXA 300 nm	Washout
R_in_ (MΩ)	629.8 ± 527.2 (10)	626 ± 520.0 (10)^ns^	462.6 ± 217.0 (8)^ns^
AP amplitude (mV)	73.69 ± 10.33 (10)	70.87 ± 9.25 (10)^ns^	70.82 ± 8.11 (9)^ns^
AP threshold (mV)	−57.74 ± 4.19 (10)	−57.03 ± 3.2 (10)^ns^	−57.91 ± 2.86 (10)^ns^
Rheobase (pA)	52.11 ± 25.07 (10)	52.54 ± 31.09 (10)^ns^	51.58 ± 27.71 (9)^ns^
Average *F*/*I* slope (pA/Hz)	0.3348 ± 0.16 (10)	0.2960 ± 0.181 (10)^ns^	0.2965 ± 0.127 (7)^ns^
Instantaneous *F*/*I* slope (pA/Hz)	0.3996 ± 0.335 (10)	0.3896 ± 0.318 (10)^ns^	0.3512 ± 0.098 (7)^ns^

Values are the mean ± SD (number of dCINs). Report of the statistical significance between control condition pre-OX and OXA at 300 nm, and the control and washout of OXA. ANOVA followed by Student–Newman–Keuls *post hoc* test was used to determine the significance between conditions.

^ns^*p* > 0.05.

### Effect of orexin on dorsal spinal cord networks

**Figure 8.**
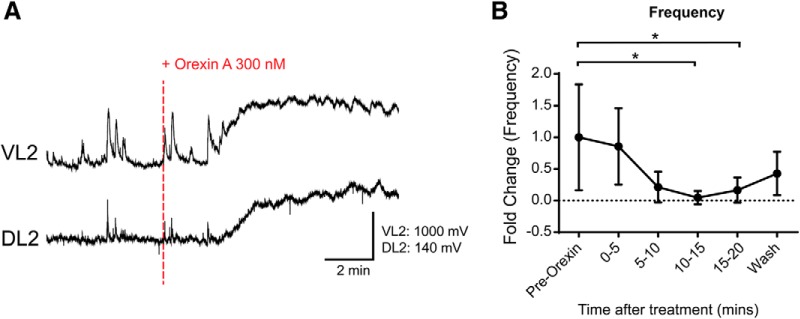
Orexin A decreases dorsal horn subthreshold potentials. ***A***, Neurogram describing the potentials generated in ventral and dorsal L2 roots. Recordings have been filtered with a Butterworth 5 Hz low-pass filter. They depict 5 min preapplication and 10 min postapplication of 300 nm OXA to an intact isolated spinal cord. ***B***, Frequency change in dorsal root subthreshold potentials taken in 5 min bins. Events were counted manually. Data are represented as the mean ± SD. *p < 0.05.

Since OXA did not produce any significant effects on dCINs and VLF projecting interneurons we next wanted to investigate whether a network of dorsally located interneurons could mediate the effects observed with OXA application. First, in intact spinal cord isolated preparations, we found that bath application of OXA, as already shown, produced an increase in tonic discharge from the L2 ventral root neurograms (*N* = 5; mean fold of change: 3.22 ± 0.77, *p* = 0.0008; data not shown). In contrast, there was no change in tonic discharge generated by the L2 dorsal root (*N* = 5; DRL2 mean fold change: 1.02 ± 0.058, *p* = 0.91). A general finding was that the frequency of subthreshold spontaneous DC neurogram activity recorded from the L2 dorsal root segments was reduced with the addition of OXA (*N* = 6; DRL2, *p* = 0.0010; Fig. 8). More specifically, there was a significant decrease in frequency in the L2 dorsal root neurogram comparing the pre-OX frequency with both the 10–15 and 15–20 min intervals after the application of OXA (*N* = 6; mean fold change in frequency from baseline to 10–15 min: 0.048 ± 0.060, *p* = 0.020; mean fold change in frequency from baseline to 15–20 min: 0.17 ± 0.089, *p* = 0.047). The decrease in the frequency of the subthreshold activity may reflect an action on GABAergic interneurons within the dorsal horn.

**Figure 9.**
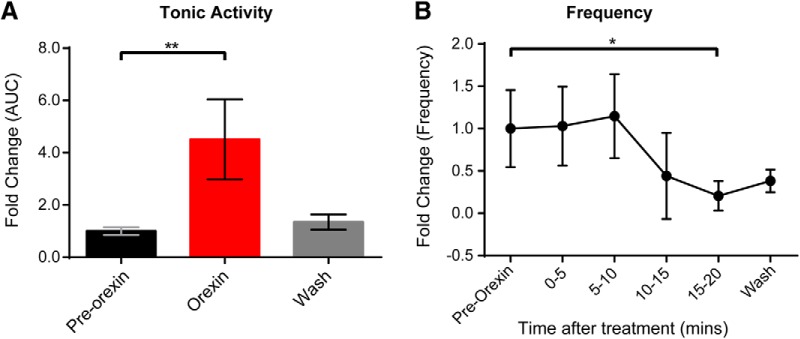
The effect of OXA on spontaneous activity in the dorsal horn-removed preparation. ***A***, OXA (300 nm) increased tonic firing activity recorded from the L2 ventral roots in the isolated ventral spinal cord preparation with dorsal horn removed (*N* = 6 dorsal horn-removed spontaneous preparations; fold change in activity). ***B***, Graph showing the decrease in spontaneous bursting activity recorded from the L2 ventral roots before and after the bath application of 300 nm OXA to a dorsal horn-removed preparation. Mean fold change in the frequency shown in 5 min bins. Data are represented as the mean ± SD. *p < 0.05, **p < 0.01.

We then used an isolated ventral spinal cord preparation with the dorsal horn removed to test the necessity of the dorsal horn network in mediating the OXA effects. In dorsal horn-removed preparations, OXA increased the tonic activity recorded from L2 ventral roots (*N* = 6; mean fold change: 4.51 ± 1.53, *p* = 0.0001; Fig. 9*A*). Additionally, in the dorsal horn-removed preparation, the application of 300 nm OXA caused a decrease in spontaneous bursting frequency, in the L2 ventral root neurograms (*N* = 6; *p* = 0.0033) at the 15–20 min postapplication time point (fold change in frequency: 0.21 ± 0.17; *p* = 0.028; Fig. 9*B*). No change in spontaneous bursting activity was detected in the ventral L5 neurograms (*N* = 6; *p* = 0.93; data not shown). These results differed from the intact isolated spinal cord preparation where OXA increased spontaneous bursting frequency (Fig. 2). Additionally, we found that it was possible to elicit fictive locomotor activity in dorsal horn-removed, isolated spinal cords following bath application of NMDA, 5-HT, and DA, supporting previous work (Dyck and Gosgnach, 2009). Subsequent bath application of OXA did not produce a change in locomotor frequency in L2 ventral roots (*N* = 5; *p* = 0.90; data not shown). These data argue for an excitatory effect from the dorsal horn on both spontaneous and fictive locomotor rhythms by OXA.

Together, these data suggest that the effects of OX on lumbar network activity are, in part, due to an action of OXA on dorsal horn interneurons, which once activated by OXs are modifying the activity (spontaneous and fictive locomotion) recorded from the ventral roots.

## Discussion

This work provides several lines of evidence that, as early as the first days of birth, OX, an important component of the motivated behavior circuit, is capable of modulating motor output in isolated spinal cord preparations. First, OXergic fibers are present in all segments of the lumbosacral spinal cord perinatally. Second, OX can modulate fictive locomotor activity and alter intrinsic motoneuronal properties. Third, leg-attached preparations show that OX can modulate EMGs from TS and TA muscles showing OX modulation of specific muscle in the hindlimbs.

### Orexin A can activate somatic motoneurons

Several lines of evidence suggest that OX can activate somatic MNs. Recording from ventral roots shows that when fast neurotransmitter blockers are applied, OXA strongly increases activity in AC and in DC recordings, reflecting population recordings of presumptive MNs leading to a sustained bursting discharge in the neurograms. Given that preganglionic neurons are known to respond to OXA in the adult rat to modulate sympathetic activity (van den Top et al., 2003), it is possible that preganglionic neurons projecting into the ventral roots at the L2 segment may have contributed to this increase in discharge from the L2 neurograms. This would be consistent with data showing an important role for orexin in the sympathetic nervous system (Carrive, 2017); however, EMG recordings also show increases in bursting when OXA was applied demonstrating that MNs innervating the limbs are recruited. Furthermore, recordings from visually identified MNs from spinal cord slices show depolarization of the resting membrane potential, accompanied by an increase in R_in_ and a shift to the left in the *F*–*I* relationship. Together, these data argue for a substantial effect of OXA on somatic MNs. While a complete examination of conductances contributing to the changes in intrinsic properties is beyond the scope of the current study, the effects of OX have been extensively studied in other areas of the brain. The increase in R_in_ could be caused by inwardly rectifying K^+^ as well as Twik-related Acid-Sensitive K^+^ channels and I_A_, respectively, being closed by OX (Hoang et al., 2004, 2003; Doroshenko and Renaud, 2009). The increase in R_in_ was not observed in adult cats following OXA microejection in the vicinity of MNs (Yamuy et al., 2004). Several reasons could account for this difference, including developmental changes affecting MN size, changes in channel expression, and the different approaches deployed (microejection vs bath application of OXA). Nonselective cation conductances, in particular transient receptor potential-canonical channels, have been implicated in the depolarizing effects of OX. In addition, Na^+^–Ca^2+^ exchangers have also been suggested to contribute by pumping out Ca^2+^ while pumping in Na^+^. Thus, several ion channel targets for OXA and OXB need to be considered. While we have no data on this topic, reports suggest that OX may have presynaptic effects on voltage-gated Ca^2+^ channels leading to an increase in the probability of transmitter release (Borgland et al., 2009). That said, afferent transmission onto dorsal horn interneurons and monosynaptic reflexes on MNs are depressed by OX (Grudt et al., 2002; Shono and Yamamoto, 2008; Jeon et al., 2015). These effects associated with the antinociceptive properties of OX suggest that OX may act to amplify inputs from premotor interneurons while depressing sensory input from the periphery. Indeed, a role for presynaptic modulation of premotor targets onto MNs should be considered since OXB can increase mEPSP frequency and cause paired-pulse depression of AMPAR currents in the VTA (Borgland et al., 2008).

### Activation of motor networks–fictive locomotor activity and spontaneous activity

Our tests of OXA function showed that in addition to effects on somatic MNs it also had a clear effect on spinal motor networks. This was especially true for spinal cords that had no other exogenous drugs applied. Here we observed increases in both the frequency and amplitude of the slow bursts as well as an increase in neurogram tonic discharge. In no case did we see OXA application evoke patterned locomotor-like activity. On the other hand, OXB application did not affect bursting but did increase tonic discharge. This suggests that OXA has a greater effect on the motor network encompassing ventral horn interneurons compared with OXB, perhaps due to its dual affinity for OX1 and OX2 receptors (Sakurai et al., 1998). While not tested here, these excitatory effects on MNs from the network could be mediated by an OXergic increase of the expression of NMDA receptors, as has been observed in the VTA (Borgland et al., 2006, 2008; Scammell and Winrow, 2011). It must also be considered that increases in MN discharge could be caused by an inhibition of GABA release. This has been observed in the VTA whereby OX1R results in retrograde 2-arachidonoylglycerol release presynaptically inhibiting GABA release via presynaptic CB1 receptors (Tung et al., 2016). Interestingly, a CB1-mediated effect on premotor neurons has been observed in spinal networks (Kettunen et al., 2005; el Manira and Kyriakatos, 2010), so that this hypothesized OX1R mechanism may act cooperatively with the mGluR1 system already proposed.

The network generating spontaneous activity is formed early in development (E5) and is characterized by synchronous bursts of activity generated throughout the rostrocaudal extent of the spinal cord (Nishimaru et al., 1996; O’Donovan, 1999; Ren and Greer, 2003; Hanson and Landmesser, 2004; Myers et al., 2005). The role is not fully understood but is causally linked to establishment and refinement of synaptic connectivity. At birth, the spontaneous activity is irregular but can produce higher-frequency rhythms when the network is sufficiently depolarized (Whelan et al., 2000). Our work provides evidence that OXRs can modulate spontaneous activity during postnatal development (van den Pol et al., 2001).

We then examined fictive locomotion evoked by bath application of 5-HT, DA, and NMDA. Under these conditions, the locomotor CPG is activated, and regular bouts of left–right alternating activity are generated. This activity differs from spontaneous activity since it is regular, the frequency is higher, and the activity resembles stepping (Whelan et al., 2000). Under these conditions, OXA produced an increase in locomotor-like frequency similar to spontaneous bursting, but no observable increase in the amplitude of bursting. The effects on the rhythm could come from the activation of a SHOX2^+^, non-V2a class of interneurons (Dougherty et al., 2013), or other interneuronal classes such as Hb9 (Hinckley et al., 2005; Wilson et al., 2005; Tazerart et al., 2008; Caldeira et al., 2017), but this remains to be determined. Overall, the effects on fictive locomotor activity were more modest. OXB had no effects on any parameter measured during fictive locomotion. One possible explanation for these differences is the changes in conductance states following the administration of locomotor-inducing drugs. During scratch-like and swimming activity in the turtle, the conductance changes twofold to fourfold over the course of a fictive episode (Alaburda et al, 2005; Guzulaitis et al, 2016). At the height of the conductance increase, many intrinsic properties typically observed in MNs are decreased. This change in conductance would translate to OXA having a reduced impact on R_in_, and this alone could partly explain the difference in effects. We have also observed differences in the effects of dopamine, which are linked to the background excitation state of the network (Sharples and Whelan, 2017). From a functional point of view, this would suggest that OXA and OXB would have greater effects in the transition between quiescence and walking rather than a sustained effect on locomotion itself. It also matches findings that OXergic activation has widespread effects throughout the CNS, which is correlated with changes in arousal and appetitive behavior (Hagan et al., 1999; Alexandre et al., 2013).

### Endogenous source of orexin A and B

Our work shows evidence of OX-IR^+^ fibers perinatally in thoracolumbar spinal cord segments, with most fibers clustered around preganglionic neurons and the dorsal horn. Of interest was the presence of OX-IR^+^ fibers that were varicosity rich. The presence of varicosities correlates with synapses but could also indicate volume transmission of OX. Volume transmission of neuromodulators within the spinal cord has been reported before (Ridet et al., 1993; Ridet and Privat, 2000; Hentall et al., 2006), and mismatches between transmitters and receptors can be accounted for by this phenomenon where neuromodulators act with long temporal windows (Jansson et al., 2002; Chrobok et al., 2017). Although we are not aware of other work in the neonatal spinal cord, in adults OX-IR^+^ fiber expression is robust across all segments of the spinal cord from cervical to sacral levels (van den Pol, 1999). In the gray matter, OX fibers terminate in all laminae. Lamina I receives higher amounts of innervation compared with lamina II and III, lamina X is strongly innervated, and in the ventral horn that houses the locomotor circuitry, OX-IR^+^ axons were present in laminae VII–IX (van den Pol, 1999). Similar to other neuromodulators, we would expect a gradual increase in OX fiber density across the first 2–3 postnatal weeks. In neonates, there is evidence of both OX1R and OX2R mRNA expression in all lamina of the spinal cord, albeit with greater expression of OX1R compared with OX2R (Allen Brain Atlas; http://mousespinal.brain-map.org/imageseries/show.html?id=100039059,100036732). These data correspond with our electrophysiological findings and mirror findings from other systems. We suspect that OXR would be present in embryonic preparations as receptor expression generally precedes fiber development. OX1/2 receptor antagonists did not modify the pre-existing spontaneous bursting, suggesting a lack of residual OXA or OXB within the isolated spinal cord. A caveat here is that our work was performed using *in vitro* tissue, with severed OX fibers.

With orexin fibers being relatively sparse around the ventral horn in the neonate, how would MNs and networks be activated? As mentioned earlier, volume transmission within the spinal cord is one possibility. Another possibility is OX innervation of the dorsal horn, since our data show a greater proportion of OX fibers in the area, and our electrophysiological data show differences in the modulation of network bursting frequency in dorsal horn-removed preparations. Furthermore, sympathetic tone likely contributes to excitability since isolated thoracic spinal segments can produce rhythmic activity (Falgairolle and Cazalets, 2007) and numerous studies suggest that T13-L2 segments, containing autonomic innervation, are especially rhythmogenic (Kjaerulff and Kiehn, 1996; Cowley and Schmidt, 1997; Kremer and Lev-Tov, 1997). Another possibility is that OX levels in the blood are relatively high in neonates (Aran et al., 2012), raising the possibility of OX rapidly crossing the blood–brain barrier (Kastin and Akerstrom, 1999). If this were to occur in neonates, it would likely be predominantly OXA that would cross since it is lipophilic, whereas OXB is lipophobic and is metabolized quickly within the blood (Nishino, 2006). Additionally, any OX released at the level of the spinal cord could be coreleased with dynorphin as dynorphin is frequently coexpressed in OX^+^ neurons and packaged within the same vesicles (Muschamp et al., 2014). Dynorphin acting via κ-opioid receptors activates the G_i/o_ signaling system inhibiting neurons. Thus, a push–pull mode of action is likely present in the spinal cord like other areas of the brain. Also, glutamate and neurotensin are reported to be coexpressed. This leads to heterogeneity of actions of OX^+^ neurons whose actions are determined by the expression of κ-opioid, glutamate, OX1/2, and neurotensin receptors (Rosin et al., 2003; Furutani et al., 2013; Muschamp et al., 2014).

### Functional implications

This article has shown a robust effect of OXA in depolarizing MNs and shows a modulatory increase in spinal network activity. OXB presumably acts at a premotoneuronal level and does not appear to directly act on MNs. Indeed, the activation of lateral hypothalamic cells containing OXergic neurons show the highest activation during motivated locomotor behavior (Torterolo et al., 2011). As such, OX may form part of a coordinated locomotor response that includes the mesencephalic locomotor region (Sherman et al., 2015) and spinal cord networks. Through a dense mesopontine projection, OX has been proposed to increase motor tone (Peyron et al., 1998; Takakusaki et al., 2016), and known OX projections to the Raphe nuclei could also indirectly influence motor function (Jordan and Sławińska, 2011). Our work shows that OX has a major effect on motoneuronal activity in neonates, especially when motor networks are less active. Our data show that OXs modulate spontaneous activity, suggesting a role of OX in the development of lumbar spinal motor circuits. Given the importance of suckling postnatally, and the role of OX in feeding, an attractive hypothesis is that OX release in the spinal cord could contribute to the priming of spinal cord networks when movement in the nest is required.

## Synthesis

Reviewing Editor: Trevor Drew, University of Montreal Decisions are customarily a result of the Reviewing Editor and the peer reviewers coming together and discussing their recommendations until a consensus is reached. When revisions are invited, a fact-based synthesis statement explaining their decision and outlining what is needed to prepare a revision will be listed below. The following reviewer(s) agreed to reveal their identity: Lea Ziskind-Conhaim. Note: If this manuscript was transferred from JNeurosci and a decision was made to accept the manuscript without peer review, a brief statement to this effect will instead be what is listed below.

The authors revised the manuscript adequately to address previous comments/concerns.

Minor suggestions:

1. Line 636: Change “..Oxergic potentiation of NMDA receptors...” to: Oxergic increase of the expression of NMDA receptors.

2. Line 659: Include Hinckley et al 2005 in the list of references linked to Hb9 interneurons.

3. Line 1000: Legend Figure 4 is misleading. The Fig doesn't show that the action of OXA is mediated by OX1R. It demonstrates the effects of OXA, an agonist that binds to both OX1R and OX2R. Specific OX1R antagonist was not used in the study.

4. Line 68: Change “are” to “is”
